# Evolution of memory system‐related genes

**DOI:** 10.1002/2211-5463.13224

**Published:** 2021-06-28

**Authors:** Amal Bajaffer, Katsuhiko Mineta, Takashi Gojobori

**Affiliations:** ^1^ Computational Bioscience Research Center (CBRC) King Abdullah University of Science and Technology (KAUST) Thuwal Saudi Arabia

**Keywords:** evolution, memory, episodic memory, long‐term potentiation, sensitization

## Abstract

Memory has an essential function in human life as it helps individuals remember and recognize their surroundings. It is also the major form of cognition that controls behavior. As memory is a function that is highly characteristic of humans, how it was established is of particular interest. Recent progress in the field of neurosciences, together with the technological advancement of genome‐wide approaches, has led to the accumulation of evidence regarding the presence and similar/distinct mechanisms of memory among species. However, the understanding of the evolution of memory obtained utilizing these genome‐wide approaches remains unclear. The purpose of this review was to provide an overview of the literature on the evolution of the memory system among species and the genes involved in this process. This review also discusses possible approaches to study the evolution of memory systems to guide future research.

## Graphical abstract

Here, Bajaffer *et al*. reviewed the evolution of a memory system and its related genes. As memory is a function that is highly characteristic of humans, how it was established is of great interest. Mechanisms and genes among species were overviewed. This review also discussed possible approaches to study the evolution of memory systems in the future.

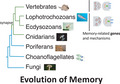

**Abbreviations**
STM: Short‐term memory
LTM: long‐term memory
GABA: gamma‐aminobutyric acid
NMDA: N‐methyl‐d‐aspartate
AMPA: α‐amino‐3‐hydroxy‐5‐methyl‐4‐isoxazole propionic acid
LTF: long‐term facilitation
WM: working memory
LTP: long‐term potentiation
E‐LTP: early‐long‐term potentiation
L‐LTP: late long‐term potentiation
STF: short‐term facilitation
AC: adenylyl cyclase
MAPK: mitogen‐activated protein kinase
IEGs: immediate early genes
C/EBPB: CCAAT enhancer‐binding protein beta
tPA: tissue plasminogen activator
BDNF: brain‐derived neurotrophic factor
5‐HT: serotonin
C/EBP: CCAAT enhancer‐binding protein
AF: activity factor
EF1alpha: elongation factor 1 alpha

Memory is the ability to store and retrieve information over time [1]. The capability to remember and recognize people, places, and things in daily life is the major form of cognition that controls behavior [2]. Thus, memory is characteristic of higher organisms. Because of its importance, memory is one of the most intensively researched subjects in the field of neuroscience. In humans, memory is one of the fundamental functions of all learning and studying processes. The importance of memory extends to child development as it helps children remember skills that they learned previously, including reading, writing, and motor skills. Nonhuman animals can also use memory for controlling their behavior in terms of social interactions, foraging behavior, and remembering predators that should be avoided in the future [3]. Therefore, it is of particular interest to understand the evolutionary process of memory systems in humans. Advancements in the field of neuroscience research have shed light on the molecular mechanisms involved in memory systems. Figure 1 shows the phylogenetic relationship of animals and the evolution of the nervous system. Ryan and Grant, 2009 [4] have addressed the evolution of the nervous system, as shown in Fig. 1; however, research on the evolution of memory is limited. In this review, we provide an overview of the evolution of memory based on genes involved in the memory system.

**Fig. 1:**
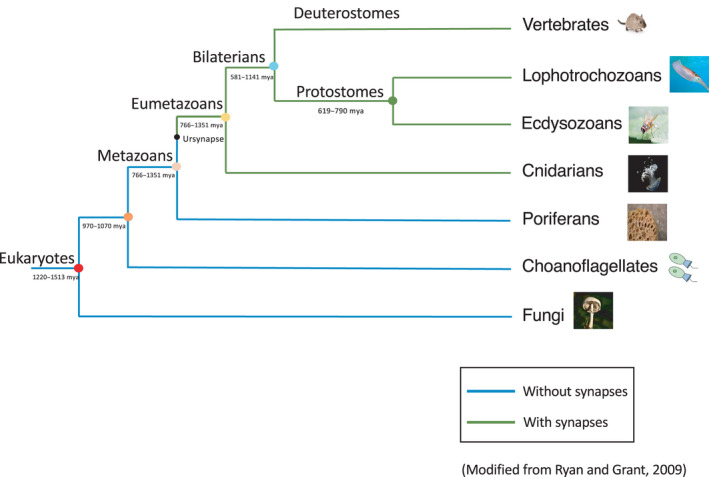
Phylogenetic relationship and evolution of the nervous system. The nodes on the phylogenetic tree indicate the points of divergence of several clades and are indicated as colored circles. The blue node represents the last common ancestor of the bilaterians. The small black circle represents the last common ancestor of synapses (ursynapse). Beside each node, the divergence time range is given in millions of years (mya). This figure was modified from Ryan and Grant, 2009 [4].

## Representative neurobiological models of memory

The memory function has three necessary stages: encoding, storage, and retrieval of information [5]. Encoding refers to the initial learning of information after perceiving it, storage is defined as the maintenance of the information over time, and retrieval refers to the ability to access and recall the information when needed [6]. According to Atkinson and Shiffrin (1971), memory involves three components: sensory register (sensory memory), short‐term memory (STM), and long‐term memory (LTM) [7]. Sensory memory is responsible for detecting information using the senses, that is, hearing, sight, taste, smell, and touch, which are directly linked to the nervous system. The storage capacity of sensory memory is large and has a short duration of few seconds [7]. STM refers to the ability to store information for a short period. The storage capacity of STM is approximately 7 ± 2 “chunks” of items [8], and it has a short duration of approximately 18–20 s [9] or 15–30 s [10]. The third type of memory is LTM. Compared with sensory memory and STM, LTM has unlimited capacity to store information and lasts for a very long time [7, 11]. At the cellular level, LTM requires gene expression (transcription), protein synthesis (translation), and the creation of a new synaptic connection, in contrast to STM, which does not require the synthesis of new proteins [12]. Neuroscientists have argued about how memory is stored in the brain and have proposed several models and theories of memory. For example, some researchers agree with the synaptic theory of memory, whereas others believe a theory known as Hebb’s theory of learning and memory. In this review, we provide an overview of both theories as being representative of memory.

### Synaptic theory of memory

In the synaptic theory of memory, memory synapses are considered as specialized synapses with a particular supplement of receptor complexes, active zone proteins, synaptic adhesion‐related proteins, and scaffold proteins, which determine the specific characteristic of the synapses that support memories [13]. Many neuroscientists agree that alterations in the strength of the synaptic connection between neurons encode memories [14, 15]. Neural diversity to express various molecules is essential for distinguishing characteristics of different neurons, and it allows variations in neuronal synapses [16, 17, 18]. In fact, various molecular complexes allow the differentiation of synapses, including gamma‐aminobutyric acid (GABA) receptor complexes [19], *N*‐methyl‐d‐aspartate (NMDA) receptors [20, 21, 22, 23], and a variety of α‐amino‐3‐hydroxy‐5‐methyl‐4‐isoxazole propionic acid (AMPA) receptor complexes [13].

Some studies have provided support for the synaptic theory of memory. Memory synapses can be erased by inhibiting reconsolidation [13]. In *Aplysia*, the use of protein synthesis inhibitors to block reconsolidation after recalling led to the loss of behavioral sensitization [24, 25]. Furthermore, memory synapses are erased with the use of pharmacological agents. Chelerythrine and zeta inhibitory peptides are pharmacological agents that were shown to erase behavioral sensitization and long‐term facilitation (LTF), increase the strength of synapses in sensory‐motor neuronal cultures in *Aplysia* [26], and disrupt long‐term potentiation (LTP) in vertebrates [27, 28, 29]. LTP is defined as the continued increase in the strength of synapses following the activation of chemical synapses [30]. Protein synthesis inhibitors have been used after learning to distinguish among different types of memory synapses experimentally. The addition of these inhibitors after learning blocks the formation of memory synapses between dentate gyrus neurons and entorhinal cortex neurons while maintaining the increase in the connections between dentate gyrus engram neurons and the CA3 area [13]. Thus, it was expected that the formation of memory synapses in these neural connection systems would be different [13].

### Hebb’s theory of memory

In 1949, Hebb proposed that the neurophysiological alterations during learning and memory occur over three stages: (a) synaptic changes; (b) cell assembly formation, known as “a set of neurons and the pathways connecting them”; and (c) a phase sequence that is the neural connection between cell assemblies [31]. An increase in the efficacy of synapses resulting in the subsequent activity of neural cell assemblies representing the primary building blocks of learning, memory, and cognitive approaches has been reported [32]. Moreover, a phase sequence formed by the connection of synapses in cell assemblies can be altered by experience. The reactivation of the sets of neurons leads to the recalling of memory stored in the altered synaptic connection [33, 34, 35].

The neurobiological basis of memory comprises a group of specific synaptic molecular and biochemical alterations, such as *de novo* protein synthesis, protein phosphorylation, upregulation of the expression of synaptic receptors, and the growth of synapses between and within cell assemblies, resulting in the efficacy of long‐term synaptic changes [36, 37, 38, 39]. It has been shown that changes in synapses are the primary step in the formation of the cell assembly and phase sequences and that together they form a memory [31].

At present, there is no conclusive evidence that memories remain at synapses [40]. In this review, we favor Hebb’s theory and discuss the underlying molecular mechanisms in the next section.

## Evolutionary perspective of memory formation

### Evolution of memory and the hippocampus

Previous studies of functional neuroimaging and patients with neurological deficits have shown that episodic memory, which is a type of LTM, crucially relies on the integrity of the hippocampus [41, 42, 43, 44] as well as cortical areas, including the prefrontal cortex and adjoining parahippocampal region [45, 46].

The hippocampus plays an essential role in storing memories in the mammalian brain [47]. It has been evolutionarily conserved and is present across various species, including mammals (such as humans, pigs, rodents, and bats) [48, 49], birds [50, 51, 52], reptiles (medial cortex) [53], and teleost fish (dorsolateral telencephalon) [53, 54]. Functional and neurobiological evidence highly suggests the existence of a homologous structure of the hippocampus across species. For example, birds possess a hippocampus, which has originated from a similar structure in mammals [51, 52, 55]. Moreover, areas that are homologous to the hippocampus are present in reptiles and teleost fish [56].

The function of the hippocampus in memory formation is evolutionarily conserved among different species. Inhibition of the formation of the hippocampus greatly impairs recognition and spatial memory in humans [57, 58] and spatial memory in rodents [59, 60]. Functionally, the mammalian hippocampus is comparable to the avian hippocampus. Lesions in the avian hippocampus also reveal disruption of spatial memory in birds [61, 62]. Similarly, they impair memory in goldfish and turtles [53]. Besides, the hippocampus, parahippocampal region, and prefrontal cortex play an essential role in episodic memory. These structures form a neural system that was believed to underlie the capacities of episodic memory in humans. However, this circuit has been detected across mammals, and a similar circuit has been found in the avian brain as well [56]. After considering structure–function similarities and the evolutionary history of episodic memory, it was hypothesized that episodic memory in humans shares the ancestral protoepisodic memory with other species, including birds and mammals. Moreover, the capability of this type of memory emerged before the divergence of mammals and reptiles [56].

### Evolution of memory in vertebrates

Working memory (WM) has been defined in three different ways: as STM employed during cognitive tasks, as attention utilization to control STM, and as multiple‐component systems that manipulate and hold information in STM [63]. Previous research has found that the primary structure of WM is homologous across all mammals [64]. Experimental work using animals has shown that the limits of WM may fall within the human range [64]. For example, a serial recall test of position was performed on a macaque monkey, which successfully remembered the first three objects in a sequence [65]. It was also shown that monkeys could follow three to four objects of food placed consecutively into one or two opaque containers and were able to differentiate between containers, including two vs. three items and three vs. four items [66]. The experiment also revealed a similar profile of latency and other impacts commonly observed in humans, implying that both species utilize a homologous mechanism of WM, with comparable limits [64]. A similar test that was conducted in horses showed that they could discriminate containers into which two or three apples had been placed and failed to differentiate between containers carrying four and six apples [67]. Therefore, the identification of three to four items most likely reflects their pure WM retention ability [64].

Episodic memory is a type of LTM that is defined as the ability to recall individual past experiences [56]. Clayton and Dickinson [68] studied memory in birds. They examined an individual’s ability to remember information about an event and its contents, such as what, where, and when. For instance, scrub jays could remember what food they stored, where it was located, and the time at which they cached it [68]. Similar evidence has been obtained for other bird species, such as magpies [69] and black‐capped chickadees [70]. The ability to remember what, where, and when regarding past events has also been demonstrated in several mammalian species, such as mice [71], rats [72, 73], pigs [74], meadow voles [75], nonhuman primates [76, 77], and humans [78, 79]. Therefore, the core characteristics of episodic memory are common among mammals and some species of birds [56]. The emergence of episodic memory has been speculated to have occurred parallel with the formation of the hippocampus, especially before the divergence of mammals and reptiles. Alternatively, it may have emerged from convergent evolution; however, additional evidence from reptiles and birds is necessary to address this alternative hypothesis [56].

### Evolution of memory among invertebrates

Studies of learning and memory have been conducted using invertebrates as well. The learning and memory process has been extensively studied in *Drosophila* (fruit flies), focusing on the genetic approach to elucidate the cellular, biochemical, and behavioral pathways underlying learning and memory [80], [81]. In addition, learning and memory processes have been studied at the molecular and cellular levels in *C*. *elegans* [82]. Furthermore, previous studies have reported the ability of *Aplysia* to form LTM [83, 84]. In addition, preliminary experiments conducted in planarians suggested that they exhibit learning and memory responses [85]. Shomrat and Levin [86] studied memory in planarians using an environmental familiarity protocol. They reported that individuals could remember a familiar environment for at least 14 days using a fully automated training apparatus. Though it should need further discussion, *Dugesia* *japonica* was suggested as one of the earlier species to possess memory.

## Molecular mechanisms and genes involved in memory

LTP is the process that is used to identify the molecular mechanisms underlying memory storage [87]. Experimental data regarding the link between memory storage and synaptic changes have revealed activity‐dependent long‐term changes in synaptic efficacy [40]. These involve LTP, which is commonly studied in mammalian synapses and expressed as changes in presynaptic and postsynaptic elements [88], as well as LTF, usually studied in invertebrate synapses and displayed as both pre‐ and postsynaptic changes [89]. LTP is a form of synaptic plasticity that is not unique to the vertebrate nervous system as it is also expressed in the nervous system of invertebrates [40]. There is a striking mechanistic similarity in LTP between invertebrates and vertebrates, suggesting that LTP was highly conserved during evolution [40]. The behavioral function of LTP has also been evolutionarily conserved [40]. For example, NMDA receptor‐dependent LTP plays a crucial role in classical conditioning at the sensorimotor synapses that mediate the defensive withdrawal reflex in *Aplysia* [90, 91]. However, no direct experimental evidence of a link has been found between LTP and learning [90]. Furthermore, the LTP of the octopus vertical lobe has been shown to be involved in LTM acquisition; nevertheless, it remains unknown whether similar cellular and molecular mechanisms drive the activity of synaptic enhancement [92].

**Fig. 2:**
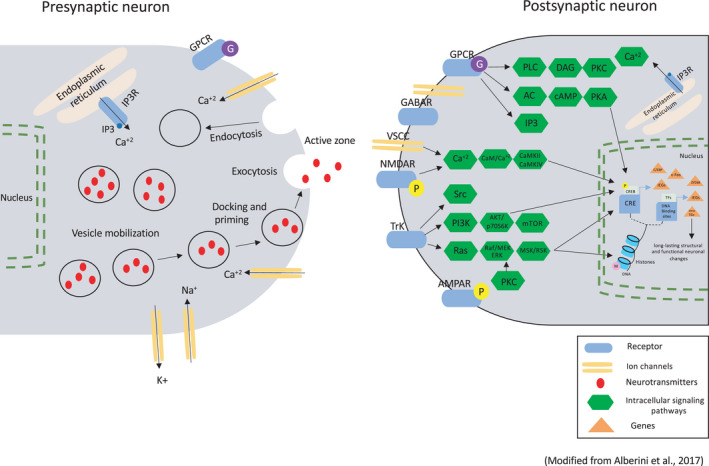
Memory system‐related genes in vertebrates and their roles. Learning induces the release of neurotransmitters such as glutamate and neuronal growth factors such as BDNF, which activate various receptor families and allow the recruitment of several intracellular signaling pathways, including second messengers and protein kinases. These signaling pathways control the following processes: 1) posttranslational modifications such as phosphorylation of postsynaptic glutamatergic receptors; 2) activation of the CREB‐mediated gene cascade, which triggers the expression of target genes such as IEGs (*Zif268*, *c‐Fos*, and *C/EBP*), thereby regulating the expression of late response genes, which are essential for long‐term structural and functional neuronal changes. The expression of these genes is regulated by many posttranscriptional and translational mechanisms, for example, the mTOR pathway, as well as by epigenetic mechanisms involving DNA methylation and histone methylation (M) and/or acetylation. This figure was modified from Alberini *et al*., 2017 [101].

Explicit spatial memory formation in the mouse hippocampus and implicit memory in *Aplysia* has common molecular mechanisms that were highly conserved during evolution [93]. Molecular and cellular studies of different types of memories have proposed that an alteration in the structure and synaptic strength is the major mechanism by which these memories are encoded and stored in the brain [93]. In fact, the storage of explicit and implicit STM requires different signaling pathways. In contrast, the storage of explicit and implicit LTM utilized common signaling pathways, such as cAMP‐response element‐binding protein 1 (CREB‐1), mitogen‐activated protein kinase (MAPK), and protein kinase A (PKA) pathways [94]. Moreover, in explicit and implicit memory, the transition from STM to LTM is regulated via inhibitory constraints [94]. Here, we provide an overview of the molecular mechanisms underlying memory in vertebrates and invertebrates (Fig. 2).

### Molecular mechanism underlying memory formation in vertebrates

The formation of explicit memory in mice occurs throughout LTP. Two stages of memory formation take place during hippocampal LTP: early LTP (E‐LTP) and late LTP (L‐LTP) [94]. E‐LTP‐like STM is produced via signal stimulation of the slice and does not require new protein synthesis [94]; instead, it induces the modification of pre‐existing proteins, leading to the modification of the pre‐existing synaptic connection, which in principle is similar to short‐term facilitation (STF) in *Aplysia* [94]. In contrast, L‐LTP‐like LTM requires multiple repetitions of stimulation, transcription, translation, and generation of new synaptic connections, similar to LTF in *Aplysia* [94].

The molecular mechanism underlying memory formation in mammalian hippocampal neurons in mice is as follows [94]. E‐LTP initiates a single train of action potentials, leading to the activation of the NMDA receptor. The influx of Ca^2+^ into postsynaptic neurons leads to the binding of the NMDA receptor to calmodulin; this complex then activates second messengers. L‐LTP is induced by repeating the trains of action potentials. The calcium/calmodulin complex binds to adenylyl cyclase (AC), which increases the cAMP level and induces PKA activation [94]. Dopamine signals can also activate AC. The catalytic subunits of PKA can stimulate MAPK and then move to the nucleus to phosphorylate CREB [94]. CREB is a transcription factor that can be activated by the PKA, MAPK, and CaM kinase pathways [95]. CREB activates the immediate early genes (IEGs, regulators, and effectors that act directly on the cell to enhance plastic changes), such as the CCAAT enhancer‐binding protein beta (*C/EBPB*), tissue plasminogen activator (*tPA*), and brain‐derived neurotrophic factor (*BDNF*) genes [94]. Protein synthesis occurs at the active synapses, leading to the growth and generation of new synapses [93].

### Molecular mechanism of memory formation in invertebrates

One form of learning in *Aplysia* is sensitization, a type of implicit memory. Sensitization refers to the learning process of responding to a stimulus in animals [94]. In a previous study, the tail shock was replaced with the application of serotonin (5‐HT), a transmitter that is usually released in animals by sensitizing stimuli [96]. A single application of 5‐HT produced STF in synaptic efficiency [93]. 5‐HT binds to the 5‐HT receptor on sensory neurons and activates the AC enzyme, which converts ATP to cAMP [94]. Subsequently, cAMP binds to the regulatory subunits of cAMP‐dependent PKA, leading to the separation of the subunits from the free catalytic subunits. These subunits can highly phosphorylate channels and induce exocytosis in the presynaptic terminals [94], leading to the reduction in K^+^ current, induction of ca^2+^ influx, increased action potential, and enhanced release of transmitters (glutamate) in sensory neurons to the follower cells [97].

In contrast, repeating the application of 5‐HT induces LTF, which can last for more than 1 week [93]. The cAMP level increases after repeated stimulation. The catalytic subunits move to the nucleus and activate MAPK. Within the nucleus, both MAPK and PKA stimulate and phosphorylate CREB and inhibit the action of CREB‐2, which is a CREB‐1 inhibitor. CREB‐1 stimulates some of the IEGs, such as an ubiquitin hydrolase that is required for regulating the proteolysis of the regulatory subunits. The cleavage of the regulatory subunits induces the persistence activity of PKA and consequently allows continued phosphorylation of PKA [94]. C/EBP is the second IEG that is stimulated by CREB‐1. C/EBP functions as a heterodimer and a homodimer together with activity factor (AF) to activate downstream genes (effector genes for growth), such as elongation factor 1 alpha (*EF1alpha*), which guide the growth of the connections of new synapses [94].

## Evolution of genes related to memory formation

As described above and in Fig. 2, many genes are involved in memory formation including (*MAPK, PKA, BDNF, C/EBP, c‐Fos, and CREB*). C/EBP proteins are characterized by the presence of a highly conserved basic‐leucine zipper (bZIP) domain [98]. Jindrich and Degnan have studied the evolution of bZIPs [99], suggesting that the bZIP family of C/EBP underwent more diversification and duplicated before bilaterian speciation. Further, BDNF was detected to be highly conserved in gene function and structure during the evolution of vertebrates and have an essential role in synaptic plasticity and during brain development [94]. In addition, Wang *et al*. [100] examined the evolution of the ATF/CREB family and found that it probably emerged in the early metazoan and expanded in vertebrate lineages. CREB controls gene regulation in response to the cAMP concentration. However, it is unclear whether CREB contributed to memory from its emergence, similar to other genes.

As memory is a system that involves many genes (Fig. 2), it is difficult to understand the evolution of the memory “system” from a single gene. In the case of CREB, it regulates so‐called IEGs such as *c‐fos* and *BDNF*. Moreover, CREB participates in the cAMP network; thus, it is linked to cAMP‐related genes such as *PKA*. Therefore, a comprehensive study of memory‐related genes is needed to understand the origin and evolution of memory.

## Conclusion

The physiological characteristics of memory have been extensively studied, indicating that the memory function is conserved among mammals, birds, reptiles, and fishes and is present even in invertebrate species. However, it is difficult to elucidate the evolution of memory based on only physiological data. We then discussed the necessity of the comprehensive research of memory‐related genes to study the evolution of the memory system. The study of molecular evolution is a promising approach to examine evolution using genes; however, evolutionary history is not uniform among genes. Therefore, it must be able to handle all genes related to memory at once. It is essential to understand the evolution of memory based on memory‐related genes because of the accumulation of genome sequences and related data. Further research on the evolution of memory focusing on a genomics approach may help understand humans and their evolution.

## Conflict of interest

The authors declare no conflict of interest.

## Author contributions

KM and TG conceived the research. AB wrote the initial draft. AB, KM, and TG edited and finalized the manuscript.
